# *Achillea fragrantissima* (Forssk.) Sch.Bip Flower Dichloromethane Extract Exerts Anti-Proliferative and Pro-Apoptotic Properties in Human Triple-Negative Breast Cancer (MDA-MB-231) Cells: In Vitro and In Silico Studies

**DOI:** 10.3390/ph15091060

**Published:** 2022-08-26

**Authors:** Nora Alshuail, Zeyad Alehaideb, Sahar Alghamdi, Rasha Suliman, Hamad Al-Eidi, Rizwan Ali, Tlili Barhoumi, Mansour Almutairi, Mona Alwhibi, Bandar Alghanem, Abir Alamro, Amani Alghamdi, Sabine Matou-Nasri

**Affiliations:** 1 Biochemistry Department, College of Science, King Saud University, Riyadh 11495, Saudi Arabia; 2 Medical Research Core Facility and Platforms, King Abdullah International Medical Research Center (KAIMRC), King Saud bin Abdulaziz University for Health Sciences (KSAU-HS), Ministry of National Guard—Health Affairs (MNGHA), Riyadh 11481, Saudi Arabia; 3 Pharmaceutical Sciences Department, College of Pharmacy, King Saud bin Abdulaziz University for Health Sciences (KSAU-HS), Riyadh 11481, Saudi Arabia; 4 Cell and Gene Therapy Group, Medical Genomics Research Department, King Abdullah International Medical Research Center (KAIMRC), King Saud bin Abdulaziz University for Health Sciences (KSAU-HS), Ministry of National Guard—Health Affairs (MNGHA), Riyadh 11481, Saudi Arabia; 5 Developmental Medicine Department, King Abdullah International Medical Research Center (KAIMRC), King Saud bin Abdulaziz University for Health Sciences (KSAU-HS), Ministry of National Guard—Health Affairs (MNGHA), Riyadh 11481, Saudi Arabia; 6 Botany and Microbiology Department, College of Science, King Saud University, Riyadh 11495, Saudi Arabia; 7 Cellular Therapy and Cancer Research Department, King Abdullah International Medical Research Center (KAIMRC), King Saud bin Abdulaziz University for Health Sciences (KSAU-HS), Ministry of National Guard—Health Affairs (MNGHA), Riyadh 11481, Saudi Arabia

**Keywords:** *Achillea fragrantissima*, carbonic anhydrase, caspase activation, mitochondrial apoptosis pathway, triple-negative breast cancer

## Abstract

The aggressive triple-negative breast cancer (TNBC) is a challenging disease due to the absence of tailored therapy. The search for new therapies involves intensive research focusing on natural sources. *Achillea fragrantissima* (*A. fragrantissima*) is a traditional medicine from the Middle East region. Various solvent extracts from different *A. fragrantissima* plant parts, including flowers, leaves, and roots, were tested on TNBC MDA-MB-231 cells. Using liquid chromatography, the fingerprinting revealed rich and diverse compositions for *A. fragrantissima* plant parts using polar to non-polar solvent extracts indicating possible differences in bioactivities. Using the CellTiter-Glo™ viability assay, the half-maximal inhibitory concentration (IC_50_) values were determined for each extract and ranged from 32.4 to 161.7 µg/mL. The *A. fragrantissima* flower dichloromethane extract had the lowest mean IC_50_ value and was chosen for further investigation. Upon treatment with increasing *A. fragrantissima* flower dichloromethane extract concentrations, the MDA-MB-231 cells displayed, in a dose-dependent manner, enhanced morphological and biochemical hallmarks of apoptosis, including cell shrinkage, phosphatidylserine exposure, caspase activity, and mitochondrial outer membrane permeabilization, assessed using phase-contrast microscopy, fluorescence-activated single-cell sorting analysis, Image-iT™ live caspase, and mitochondrial transition pore opening activity, respectively. Anticancer target prediction and molecular docking studies revealed the inhibitory activity of a few *A. fragrantissima* flower dichloromethane extract-derived metabolites against carbonic anhydrase IX, an enzyme reported for its anti-apoptotic properties. In conclusion, these findings suggest promising therapeutic values of the *A. fragrantissima* flower dichloromethane extract against TNBC development.

## 1. Introduction

Breast cancer is the first-rank diagnosed cancer worldwide and the second leading cause of cancer mortality in the female population, responsible for about 685,000 deaths in 2020 [1]. Due to its heterogeneity in morphological, molecular, and biochemical features, breast cancer is one of the most challenging diseases among all types of cancer [2]. A highly aggressive subtype of breast cancer is triple-negative breast cancer (TNBC), which is mainly characterized by the lack of the expression of the estrogen receptor, progesterone receptor, and human epidermal growth factor receptor 2 [3]. This TNBC subtype, accounting for 10–20% of all breast cancer subtypes, is mostly diagnosed at the advanced stage of the cancer, and contributes to the metastatic disease, which is the most critical condition reducing patient survival [4,5]. The treatment of this heterogeneous TNBC presents a clinical challenge due to the lack of conventional targeted therapies, requiring the development of tailoring treatment, including immune-checkpoint inhibitors and antibody–drug conjugates [5,6]. Although both chemotherapy and surgery can be beneficial, many studies demonstrate that surgical removal of the cancer generates tumor relapse and metastatic recurrence [7]. Thus, chemotherapy remains the first-line standard therapeutic option, paving the way for different systemic therapies, including the development of novel anticancer natural product-based drugs [8,9,10,11].

Traditional herbal medicine and derivative natural compounds have been widely studied for their anticancer properties via different mechanisms leading to cancer cell death, including programmed cell death (i.e., apoptosis) [12,13]. Apoptosis is a complex series of proteolytic network reactions distinguished into two molecular pathways, death receptor-dependent (extrinsic) and mitochondria-dependent (intrinsic) apoptosis involving initiators caspase-8 and caspase-9, respectively [14]. Mitochondria play a regulatory role in the intrinsic apoptosis pathway through the expression of mitochondrial anti-apoptotic proteins, such as Bcl-2, and pro-apoptotic protein members of the Bcl-2 family such as pro-apoptotic proteins Bak (Bcl-2 antagonist or killer) and Bax (Bcl-2-associated X protein). Bax proteins generate oligomerization or heteromerization between Bax/Bak proteins to form large pores in the outer membrane of the mitochondria leading to the increase in outer-membrane permeabilization, which subsequently release cytochrome *c*, a main component of caspase-9 activation in the apoptosome [14]. The cleavage of these initiator caspases results in the cleavage of executioner caspases (caspase-7, caspase-3), which leads to membrane lipid profile reversion characterized by phosphatidylserine exposure, cytoskeletal and nuclear protein degradation, and deoxyribonucleic acid (DNA) degradation, causing the apoptotic body formation [15,16].

The *Achillea* genus belongs to the family *Asteraceae* (i.e., *Compositae*), which contains over 130 perennial species found in temperate climates in dry or semi-dry habitats, including the species of *Achillea fragrantissima* (Forssk.) Sch.Bip (*A. fragrantissima*) (Synonym, *Santolina fragrantissima* Forssk.), which are known as traditional herbal medicine consumed by Middle Eastern populations [17,18]. It is commonly referred to as Qaisoom (in Arabic, قيصوم) in the Middle East region [19]. This traditional herbal medicine is used to treat numerous illnesses including respiratory diseases, eye infections, smallpox, fever, gastrointestinal disturbances, dysmenorrhea, headache, fatigue, and diabetes [18,20]. In this study, we hypothesize that this understudied local traditional herbal medicine might contain phytochemical(s) that could be potential therapeutic agent(s) against TNBC.

## 2. Results

### 2.1. Screening of the Anti-Proliferative Potential of A. fragrantissima Extracts Derived from Flowers, Leaves, and Roots against TNBC Cells

**Figure 1:**
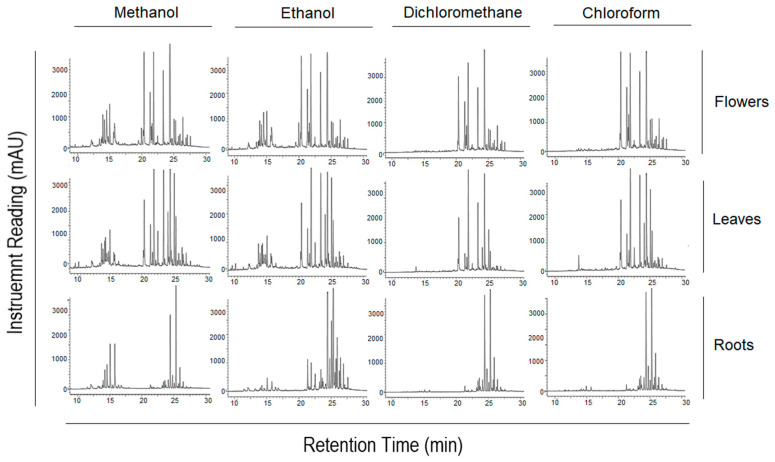
Fingerprinting chromatograms of *A. fragrantissima* flower-, leaf-, and root-derived solvent extracts using HPLC-UV. The chromatograms are based on a gradient method of methanol and water, and UV set at 275 nm with 5 µL injection volume. The separation of the dimethyl sulfoxide (DMSO)-dissolved compounds was performed using a Kinetex^®^ C-18 column (250 × 4.6 mm, 5 µm) and revealed diverse polar and non-polar compositions for the different solvent extracts of the herbal medicine.

In the search for the discovery of new anticancer natural compound-based drugs for the treatment of TNBC, different parts (i.e., flowers, leaves, and roots) of the plant *A. fragrantissima* were extracted using various solvents (i.e., methanol, ethanol, dichloromethane, and chloroform). Each aqueous *A. fragrantissima* extract was then tested for endotoxin contaminants, which was confirmed by the low endotoxin unit (EU) levels reaching 0.0032 EU/µg herb weight. Concerning the chemical analysis, the fingerprinting for each extract was analyzed using high-performance liquid chromatography and ultra-violet detector (HPLC-UVD), which displayed different chromatograms, as seen in Figure 1.

**Figure 2:**
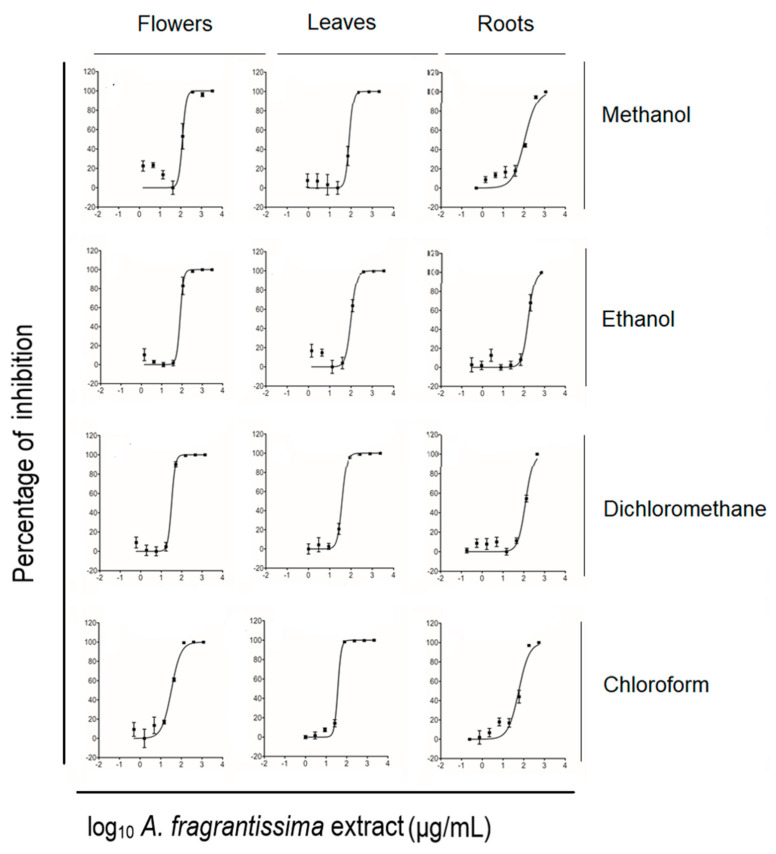
Inhibition of TNBC MDA-MB-231 cell viability by different solvent extracts of *A. fragrantissima* plant parts using the CellTiter-Glo™ assay.

The measurement of half-maximal inhibitory concentrations (IC_50_) was performed using an ATP-based viability assay. The dichloromethane extract of the *A. fragrantissima* flowers displayed the lowest IC_50_ value followed by the chloroform extract. This dichloromethane extract was used for the remaining in vitro investigations for anticancer properties. Based on the ATP generated by living untreated and treated TNBC MDA-MB-231 cells, the IC_50_ value reflecting the anti-proliferative potential of each extract was determined. All the *A. fragrantissima* extracts significantly inhibited MDA-MB-231 cell viability in a dose-dependent manner (Figure 2). The mean IC_50_ values ranged from 32.4 to 161.7 µg/mL, including the dichloromethane and chloroform extracts derived from the flowers showing the lowest mean IC_50_ values, followed by the leaf-derived extracts, as seen in Table 1. The dichloromethane extract of the *A. fragrantissima* flower was shown to be the most potent leading to MDA-MB-231 cell growth inhibition and, thus, was selected for further experiments in this study.

### 2.2. Induction of Apoptosis in MDA-MB-231 Cells by A. fragrantissima Flower Dichloromethane Extract

**Figure 3:**
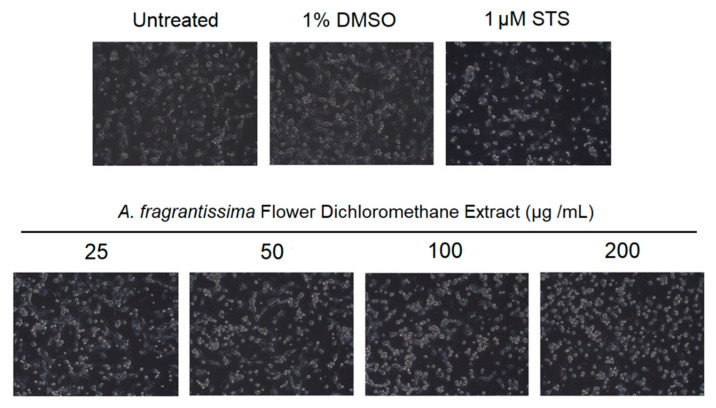
Microscopic images of the MDA-MB-231 cells treated with increasing concentrations of *A. fragrantissima* flower dichloromethane extract displaying apoptosis-related morphological changes in cell shrinkage. The MDA-MB-231 cells display a dose-dependent increase in cell shrinkage, a morphological characterization related to apoptosis. The cell shrinkage that occurred in the MDA-MB-231 cells treated with 200 µg/mL of *A. fragrantissima* flower dichloromethane extract was similar to the cells treated with the strong apoptosis inducer STS, the positive control.

**Figure 4:**
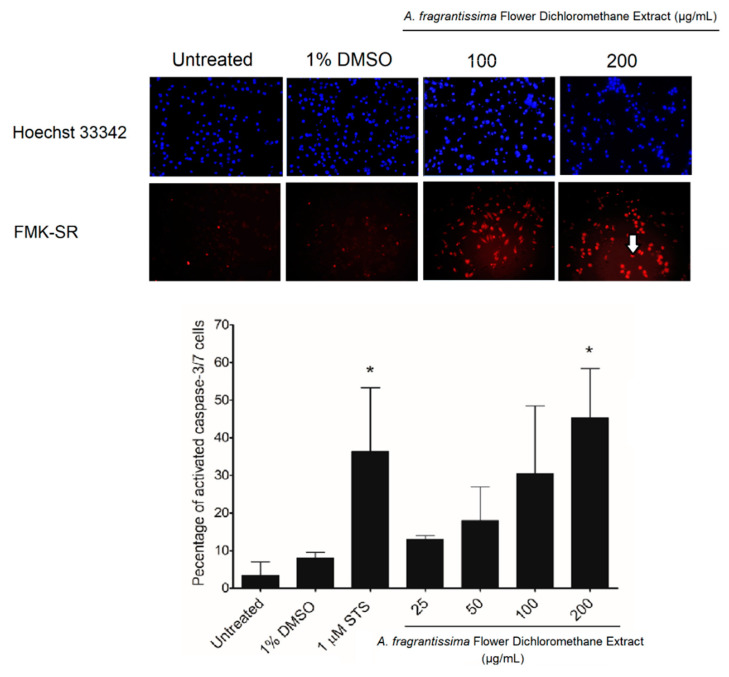
The increase in caspase-3/7 activation in the *A. fragrantissima* flower dichloromethane extract-treated TNBC MDA-MB-231 cells using confocal microscopy. The MDA-MB-231 cell treatment with the dichloromethane *A. fragrantissima* flower dichloromethane extract (25–200 µg/mL) and STS (1 µM) showed an increase in caspase-3/7 activity by more than 1.5-fold in comparison to the DMSO-treated cells, the negative control. The arrow is an example of MDA-MB-231 cells with activated caspase-3/7 (red fluorescence). Hoechst 33342 and fluoromethyl ketone-sulforhodamine (FMK-SR) were used as a nuclear dye and a cell-permeant red fluorescent probe labeling active caspase-3/7, respectively. * *p* < 0.05 vs. DMSO-treated cells.

**Figure 5:**
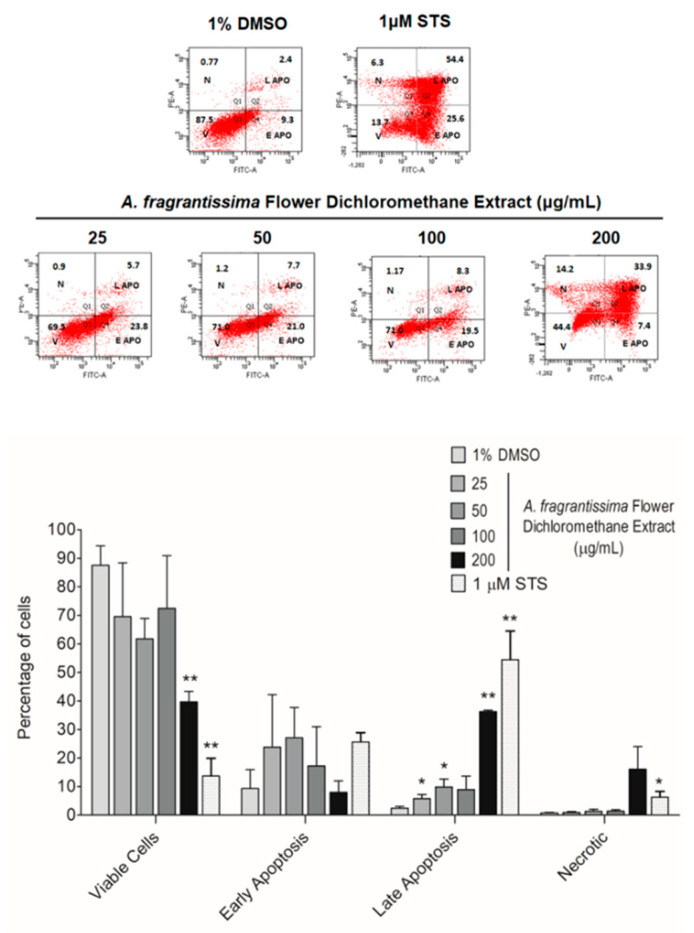
Determination of the apoptotic status in the TNBC MDA-MB-231 cells exposed to *A. fragrantissima* flower dichloromethane extract. Representative scatter plots indicating the percentage of MDA-MB-231 cells in viability (V), in apoptotic status including early apoptosis (E APO) and late apoptosis (L APO), and in necrotic (N) status, based on Annexin V (-FITC) and PI (-PE) double staining measured by FACS. *A. fragrantissima* flower dichloromethane extract prompted apoptosis in TNBC MDA-MB-231 cells, in a dose-dependent manner, compared to DMSO-treated cells. STS was used as a positive control. The bar graph presents data as mean ± SD of three separate experiments. * *p* < 0.05 and ** *p* < 0.01 vs. DMSO-treated cells.

**Figure 6:**
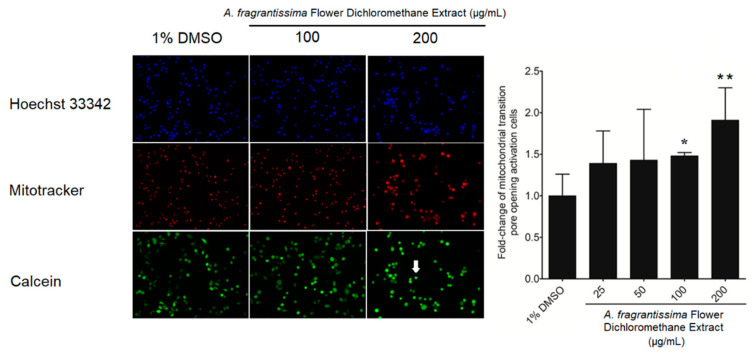
The increase in mitochondrial membrane potential opening activity in *A. fragrantissima* flower dichloromethane extract-treated TNBC MDA-MB-231 cells using confocal microscopy. The MDA-MB-231 cell treatment with *A. fragrantissima* flower dichloromethane extract (25–200 µg/mL) showed an increase in the activity of the opening of the mitochondrial transmission membrane pores, as indicated by the arrow. * *p* < 0.05 and ** *p* < 0.01 vs. DMSO-treated cells.

In vitro investigations were performed to determine if the inhibition of MDA-MB-231 cell growth was mediated via the caspase-dependent extrinsic apoptotic pathway. The treatment of MDA-MB-231 cells by the *A. fragrantissima* flower dichloromethane extract increased observed cell shrinkage in a dose-dependent manner, suggesting apoptosis as the mechanism of cell death (Figure 3). The caspase-dependent apoptosis was confirmed in *A. fragrantissima* flower dichloromethane extract-treated MDA-MB-231 cells presenting cleaved caspase-3/7; the active form indicated in red fluorescence and was determined under confocal fluorescence microscopy (Figure 4). The confocal images clearly display an increase in caspase-3/7 activity in the cells treated with the protein kinase inhibitor staurosporine (STS, a potent apoptosis inducer) and *A. fragrantissima* flower dichloromethane extract (25 to 200 µg/mL), in comparison with the untreated and DMSO-treated cells, the negative controls. The status of apoptosis was also determined using the FACS analysis based on the Annexin V/propidium iodide (PI) stain, which clearly showed early and late apoptotic status of *A. fragrantissima* flower dichloromethane extract-treated cells, in a dose-dependent manner, compared to DMSO-treated cells (Figure 5). The mitochondria potential membrane assay detected an increase in the number of apoptotic whole-cell green fluorescence indicating the involvement of the intrinsic apoptotic pathway in STS- and *A. fragrantissima* flower dichloromethane extract-treated cells, compared to the untreated and DMSO-treated cells (Figure 6).

### 2.3. Metabolite Identification Using LC-QTOF

**Figure 7:**
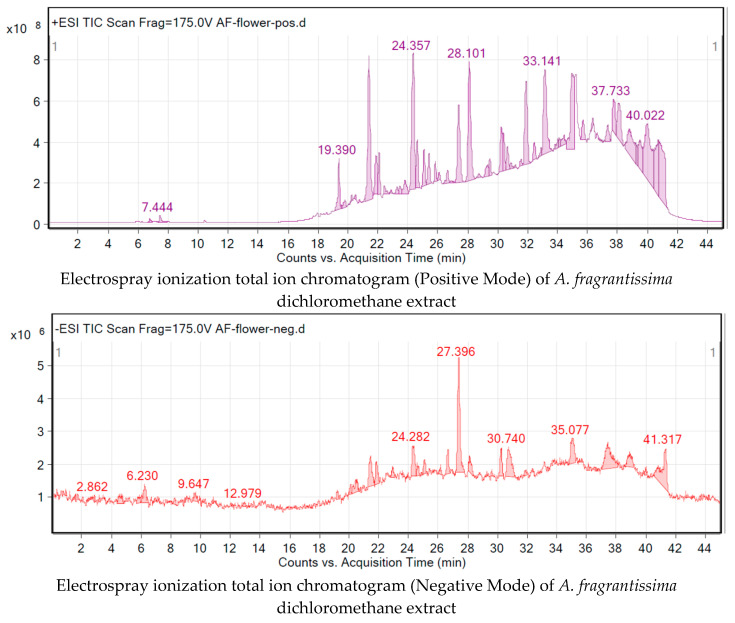

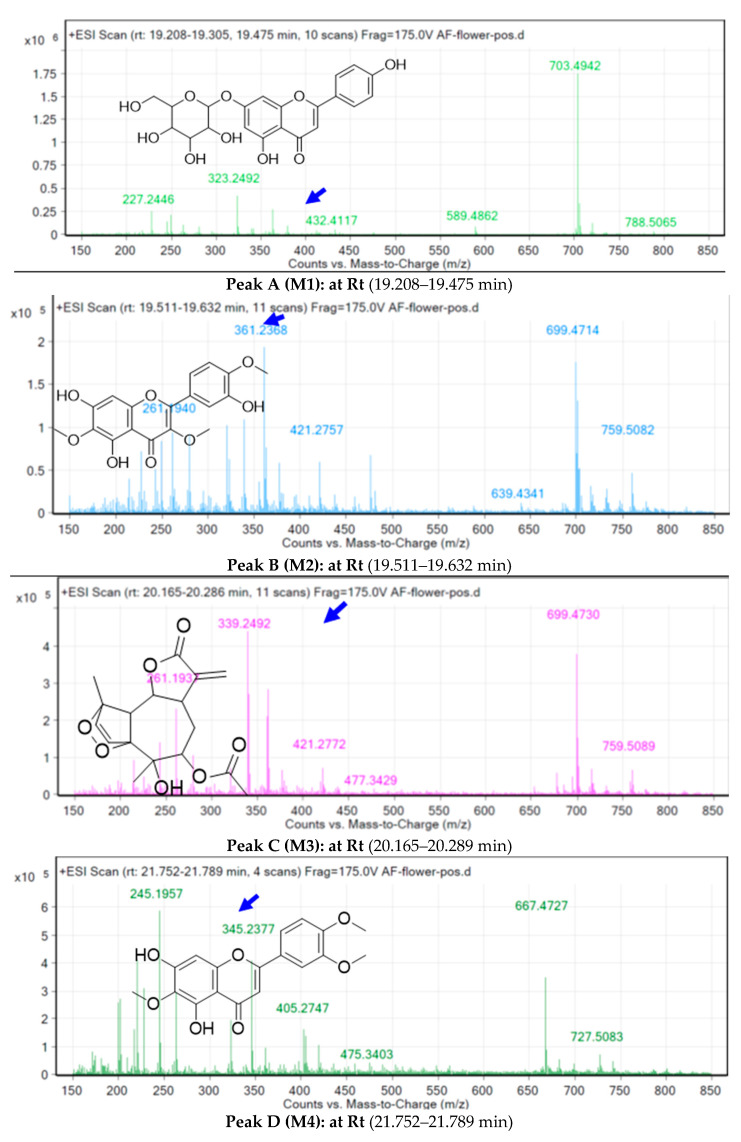

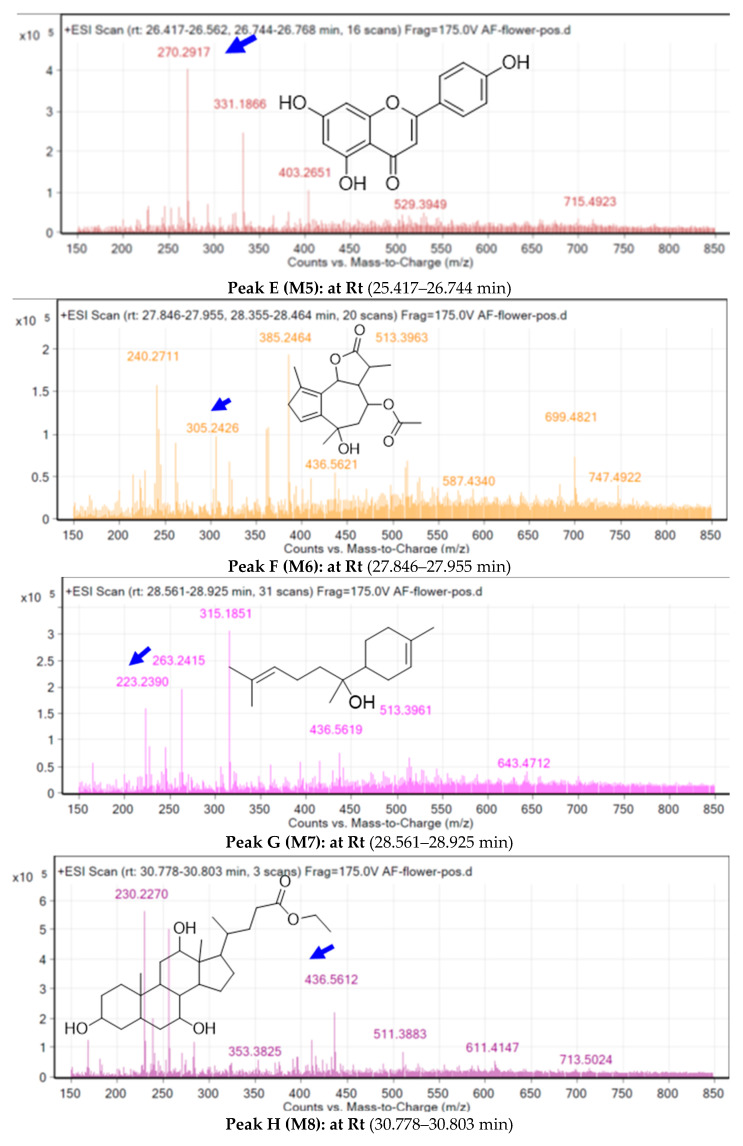

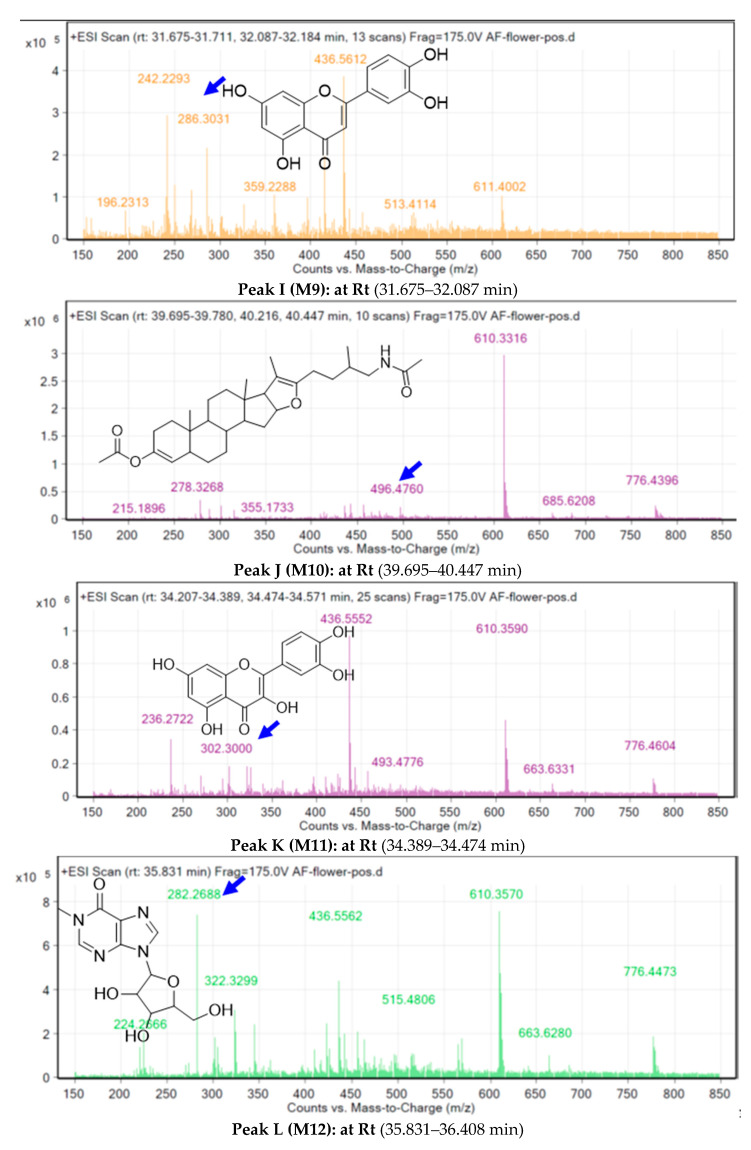

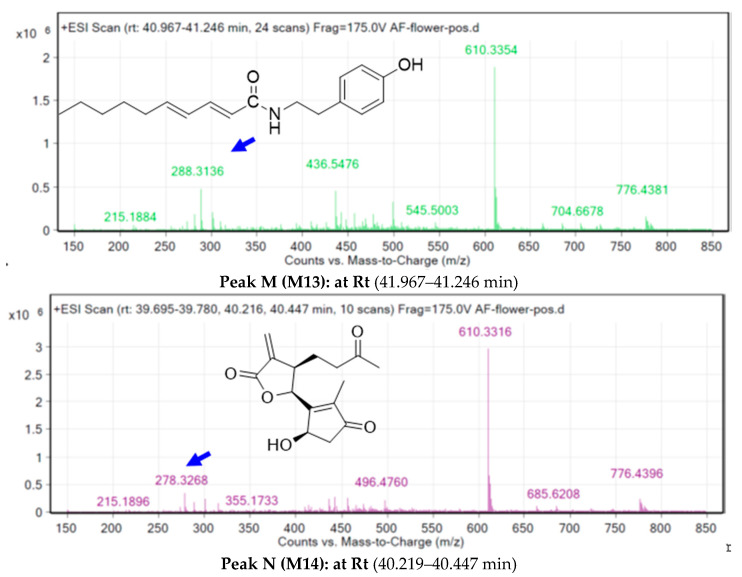
The identification of *A. fragrantissima* flower dichloromethane extract metabolites using an Agilent LC-QTOF in positive and negative modes.

The crude extract of the *A. fragrantissima* flower dichloromethane was subjected to total ion current (TIC) spectra raw data and the data analysis program MassHunter (Agilent Technologies) qualitative and quantitative analysis software was used. After conducting a mass screening on the below spectrum, chemical features were extracted from the liquid chromatography–mass spectrometry (LC-MS) data using the Molecular Features Extraction (MFE) algorithm and the recursive analysis workflow (Figure 7). Features were extracted by screening the detected nodes at various retention times (Rt) per minute, with a minimum intensity of 6000 counts, and aligned with previously detected compounds considering adducts ([M + K]^+^, and [M − H]^−^). Cosmosiin [21], Centaureidin [22], Apressin [22], Eupatilin [23], Lupeol [17], Oleic acid [24], Bisabolol, [22], Ethyl isoallocholate [25], Luteolin [26], Pseduosolasodine diacetate [25], Quercetin [27], Methylinosine [25], p-hydroxy-phenethylamine IV [28], and Tanaphillin, [29], Means *m*/*z* implies measured *m*/*z*.

### 2.4. PASS Online Anticancer Predictions

The anticancer activity of the identified metabolites in *A. fragrantissima* flower dichloromethane extract was predicted using the prediction of activity spectra for substances (PASS) online webserver. If the P_a_ value is greater than 0.3, it indicates a high probability that the metabolite will be active experimentally. As summarized in Table 2, all the 14 identified metabolites demonstrated high probabilities that were greater than 0.3 (**M5**, **M7**, **M8**, **M10**, **M12**, and **M13**), and some metabolites (**M1**–**M4**, **M6**, **M9**, **M11**, and **M14**) were higher than 0.7 indicating a high probability that these metabolites would be active experimentally. Moreover, the inactivity scores determined for the metabolites were near to zero suggesting their promising anticancer activity.

### 2.5. Molecular Target Predictions

The possible molecular targets for the identified metabolites were predicted using the Molinspiration webserver. The webserver gives an activity prediction involving main five targets important for biological effects including the G protein-coupled receptor (GPCR), ion channel, kinase, nuclear receptor, protease, and enzyme. Our results showed that most of the metabolites demonstrated high bioavailability scores ranging from 0.13 to 0.81 as enzyme inhibitors (Table 3). Moreover, some of the metabolites exhibited high bioavailability scores as nuclear receptor ligands ranging from 0.01 to 0.81. These results suggest that the observed biological anticancer activity could involve several interactions and mechanisms.

### 2.6. Molecular Docking

**Figure 8:**
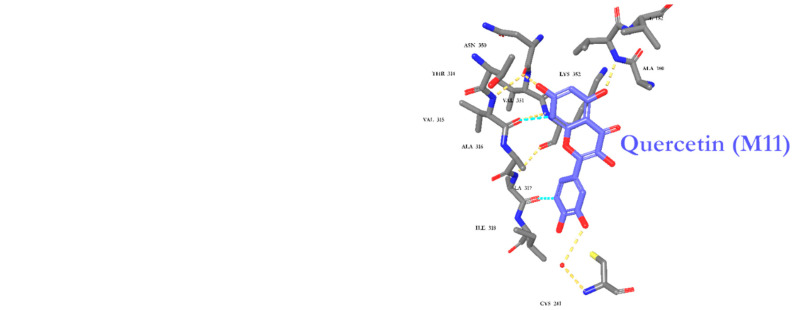

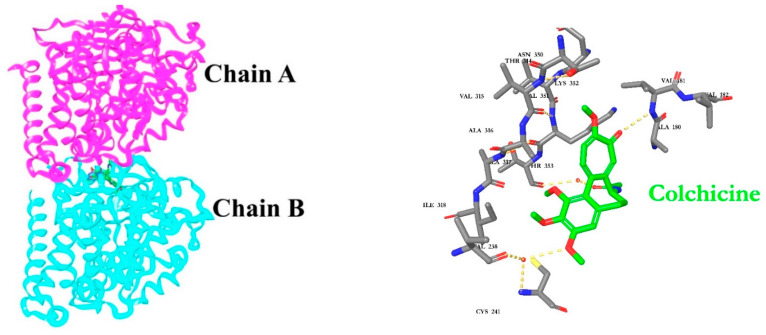
Docking poses for metabolite 11 (**M11**) and Colchicine into Tubulin crystal structure.

The fourteen identified metabolites from *A. fragrantissima* flower dichloromethane extract were docked into Tubulin [30,31,32] and Carbonic anhydrase (CA) IX enzyme [33,34], two main substrates reported to be inhibited by flavonoids, to investigate the molecular interactions using Maestro Schrödinger software. The crystal structures of Tubulin and CA IX enzyme were optimized and minimized, followed by grid generations for both binding pockets. For Tubulin docking, Colchicine (native ligand) was used as a positive control and the docking protocol was validated by docking the Colchicine into the binding site with a low root-mean-square deviation (RMSD) value relative to crystal structure. The docking scores for the fourteen identified metabolites range from −4.23 to −9.89 kcal/mol. As shown in Table 4, metabolite 11 (**M11**, Quercetin) demonstrated the highest docking score (−9.89 kcal/mol), followed by **M1** (Cosmosiin, −9.59 kcal/mol) and **M2** (Centaureidin, −9.16 kcal/mol). Moreover, **M11** exhibited several interactions with the binding site including Cys241, Val181, and Asn350, and occupied a similar binding pose relative to Colchicine as illustrated in Figure 8. All the 2-D interaction maps for the metabolites with Tubulin are presented in Figure 8.

**Figure 9:**
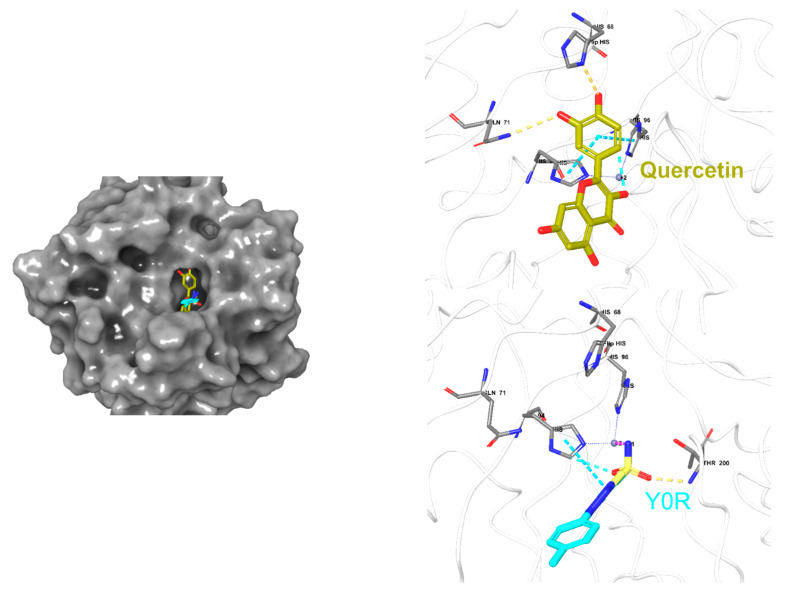
Docking poses for metabolite 11 (**M11**) and Y0R into CA IX crystal structure.

Moreover, the fourteen metabolites were also docked into the CA IX crystal structure and docking scores are summarized in Table 5. The docking scores ranged from −1.30 to −5.46 kcal/mol, and zinc coordination was maintained in the most docked metabolites. Interestingly, **M11** demonstrated the highest docking score (−5.46 kcal/mol) followed by **M4** (−5.25 kcal/mol) and **M5** (−5.21 kcal/mol). Additionally, several hydrophilic and hydrophobic interactions were formed at the binding site as depicted in Figure 9. All the 2-D interaction maps for the metabolites with CA IX are depicted in Figure 9.

### 2.7. Absorption, Distribution, Metabolism, and Excretion (ADME) Predictions

**Figure 10:**
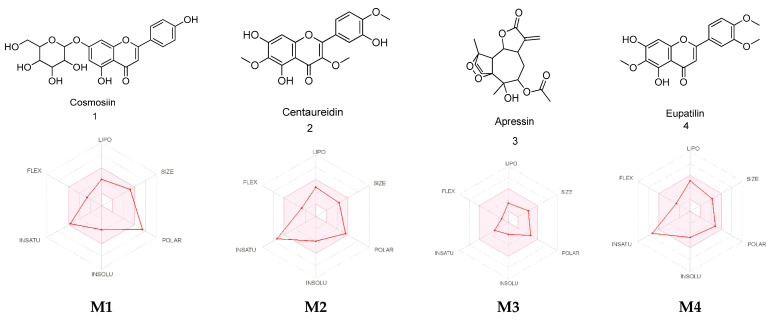

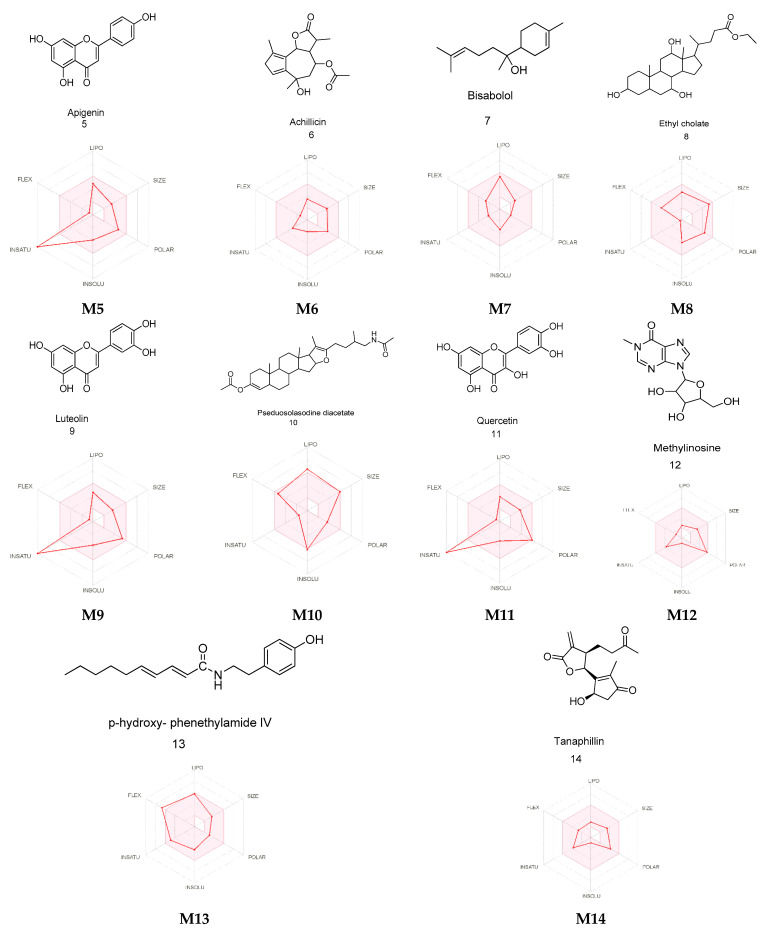
Radar diagrammatic representation of *A. fragrantissima* flower dichloromethane extract-derived identified metabolites (**M1**–**M14**) using SwissADME webserver. LIPO: Lipophilicity, Size: Molecular weight, POLAR: solubility, INSOLU: insolubility, INSATU: insaturation, and FLEX: flexibility. The properties involved in the colored zone are preferred for orally active drugs.

Several pharmaceutical properties important for drug discovery were calculated for the identified metabolites using the SwissADME web server and QikProp tool. These parameters include Molecular Weight (g/mol), Log Po/w (lipophilicity), Log S (solubility), blood–brain barrier (BBB) Permeability, and gastrointestinal (GI) Absorption. As shown in Table 6, all the metabolites exhibited acceptable pharmaceutical parameters (Figure 10) that were within Lipinski’s rule of five (ROF), except for metabolites **M1** and **M10** that violated the hydrogen bond donor/acceptor rule and molecular weight, respectively. Moreover, most of the metabolites were predicted to act peripherally with no central nervous system effects, except for **M6**, **M7**, and **M13**. Additionally, the majority of the identified metabolites were predicted to possess a high GI absorption, except for **M1** and **M12**.

### 2.8. Cytochrome P450 (CYP) Enzyme Inhibition Profiling

The effects of the identified metabolites of *A. fragrantissima* flower dichloromethane extract on CYP isoenzymes were reported in Table 7 using the SwissADME webserver. Six out of fourteen of the evaluated metabolites were predicted to inhibit CYP1A2 and CYP3A4, while five metabolites were predicted to likely inhibit CYP2D6 and CYP2C9, and only one metabolite was predicted to inhibit CYP2C19, as summarized in Table 7.

### 2.9. Organ and Endpoint Toxicity Predictions

The toxicity of the identified metabolites was assessed using the ProTox-II webserver against hepatotoxicity, carcinogenicity, immunotoxicity, mutagenicity, and cytotoxicity. As summarized in Table 8, all the metabolites were inactive as hepatotoxic and cytotoxic. While six (**M2**–**M4**, **M6**, **M8**, **M10**, and **M13**) out of fourteen were predicted to be immunotoxic, fewer were predicted to exhibit mutagenicity (**M1**, **M3**, **M9**, and **M11**) and carcinogenicity (**M6**, **M9**, and **M11**).

## 3. Discussions

Natural products, including bioactive molecules from plant, animal, fungal, and microbial origins, remain a vital source for therapeutic drug discovery. Since the last century, approximately more than fifty percent of pharmaceutical drugs for humans have been derived from natural sources [35,36]. The importance of natural products as a source of cancer treatment is well recognized and documented. It is estimated that forty-nine percent of approved small molecules for cancer treatment are derived from natural products in the time period between the 1940s and 2014 [37]. Endemic species of plants in the Middle Eastern region are among the least studied species and could potentially reveal novel lead compound(s). The medicinal plant species belonging to the *Achillea* genus found in the Middle East region are popular treatments for several illnesses, including respiratory diseases, fever, gastrointestinal disturbances, headache, fatigue, and diabetes [17,18,20]. This study explored one local species of *A. fragrantissima* for new therapies against TNBC using a series of in vitro experiments to determine its potential anti-proliferative, cytotoxic, and pro-apoptotic properties.

The investigation of this current study was initiated by chemical fingerprinting for the three plant parts of *A. fragrantissima* (i.e., flowers, leaves, and roots) using methanol, ethanol, dichloromethane, and chloroform. The solvents were chosen to represent the polar (i.e., methanol and ethanol), moderately polar (i.e., dichloromethane), and non-polar (i.e., chloroform) extractions that were reflected in the chromatograms. The chromatograms of methanol and ethanol extracts displayed more polar phytochemical constituents, which eluted first due to the separation using the reverse-phase C18 column while the least polar constituents appeared more abundant and eluted last in the chromatograms of dichloromethane and chloroform extracts. The numerous and abundant phytochemical constituents in the DMSO-reconstituted extracts also indicated the efficiency of the extraction method using the high-power probe sonicator, and that the abundant phytochemical constituents were retained after reconstitution with DMSO solvent. The rich phytochemical compositions observed in the chromatograms were consistent with studies that isolated and characterized numerous phytochemical constituents of *A. fragrantissima* plant’s parts using various techniques and identified several families of phytochemicals, including phenolic acids, flavonoids, lignans, terpenic lactones, and alkamides [18].

The anti-proliferative effects of *A. fragrantissima* plant parts-derived extracts on TNBC MDA-MB-231 cells were determined using the CellTiter-Glo™ assay. The bioassay determined the viability of cell populations by measuring the amount of adenosine triphosphate (ATP) generated by metabolically active cells. It is known that the bioassay is among the most sensitive cell viability bioassay in comparison to tetrazolium reduction, resazurin reduction, and protease activity bioassays [38]. The performance of the bioassay in this study was shown to be sensitive enough to establish the concentration–inhibition curves efficiently despite the relatively low cell seeding count. Furthermore, the variation in the inhibition concentrations values did reflect the chromatograms of the polar extracts displaying higher IC_50_ values in comparison to the least polar extracts, suggesting that the least bioactive polar compounds contributed to the weight of the extracts, resulting in higher inhibition values. The inhibition concentrations were used as a guideline to select the most potent anti-proliferative extract. The *A. fragrantissima* flower dichloromethane extract presenting the lowest mean IC_50_ value was chosen for further cell death-related molecular mechanism investigation.

Furthermore, additional confirmatory experiments were required to confirm apoptosis as the main mechanism of cell death [39]. Further experiments in this study include the observation of apoptosis-related cell morphological changes, the detection of caspase-3/7 activation, and abnormal exposure of phosphatidylserine on the outer cell membrane in treated MDA-MB-231 cells. The treatment of MDA-MB-231 cells with *A. fragrantissima* flower dichloromethane extract clearly led to a gradual increase in cell shrinkage, in a dose-dependent manner, with the highest concentrations (i.e., 100 and 200 µg/mL) displaying similar observations as the apoptosis inducer, STS, used as a positive control. For caspase-3/7 activation, the detection was based on the fluorescent inhibitor of caspases (FLICA) methodology. The method was based on the covalent binding of fluoromethyl ketone (FMK) moiety to the center of the activated caspase by a recognition sequence for capase-3/7 (i.e., aspartic acid–glutamic acid–valine–aspartic acid) [40]. The FLICA method allowed direct visualization of activated caspase-3/7 in treated MDA-MB-231 cells as red-fluorescence cells while cells without activated caspases display the nucleus only after use of the Hoechst 33342 (blue) staining. The *A. fragrantissima* flower dichloromethane extract clearly increased the number of activated caspase-3/7 cells to levels comparable to the protein kinase inhibitor, STS. The FACS analysis using the fluorescein isothiocyanate (FITC)-labeled Annexin V/phycoerythrin (PE)-labeled PI dual staining allowed the detection of viable, early apoptosis, late apoptosis, and dead/necrotic cell populations in MDA-MB-231 cells treated with STS or different *A. fragrantissima* extract concentrations. The experiment confirmed the occurrence of the early and late apoptotic status by Annexin V binding to the exposed phosphatidylserine on the outer leaf of the cell membrane, which is a hallmark characterization of programmed cell death [41]. Furthermore, the PI binding to nucleic acids is used to detect cells with lost cell membrane integrity as seen in late apoptotic and dead/necrotic cell populations, which is also considered an irreversible cell death status [42].

After confirming that caspase-dependent apoptosis as a cell death mechanism occurred in the MDA-MB-231 cells exposed to *A. fragrantissima* flower dichloromethane extract, a deeper investigation was conducted to determine the involvement of the intrinsic apoptosis pathway using the mitochondrial permeability transition pore activation assay. Indeed, apoptosis occurs in two pathways referred to as intrinsic and extrinsic. The intrinsic apoptosis pathway involves mitochondrial permeability transmembrane pore opening forming the apoptosome leading to caspase-9 activation, then subsequently to executioner caspases-3/7 activation [43]. The bioassay directly measured the mitochondrial permeability transition pore opening activity using acetoxymethyl ester of calcein dye, which accumulated in the cytosolic compartments and liberated into a highly-fluorescent calcein. In pro-apoptotic conditions, the cells with activated mitochondrial permeability transition pore opening appeared brightly green due to the redistribution of calcein from the cytosol into the mitochondria. The addition of the quencher, CoCl_2_, removed the remaining cytosolic fluorescence, while the mitochondrial fluorescence was maintained visible under the confocal microscopy. The treatment of MDA-MB-231 cells with *A. fragrantissima* flower dichloromethane extract increased the number of cells displaying activated mitochondrial permeability transition pore opening in a dose-dependent manner, confirming the involvement of the mitochondria-dependent intrinsic apoptosis pathway. Interestingly, our findings characterizing the main hallmarks of apoptosis, including caspase-3/7 detection, apoptosis status determination by FACS, and mitochondrial membrane transition pore opening activation, exhibited a similar pattern. The results clearly displayed an induction of apoptosis in TNBC MDA-MB-231 cells, which slightly and gradually increased in response to the 25 and 50 µg/mL treatment to reach the highest pro-apoptotic changes in response to 100 and 200 µg/mL of *A. fragrantissima* flower dichloromethane extract.

Numerous studies have reported the anticancer potencies of many species belonging to the *Achillea* genus, including the widely-consumed native North American species of *A. millefolium* [44]. Only a few studies reported *A. fragrantissima* crude extracts with anticancer properties using cancer cell lines. For instance, Sathiyamoorthy et al. tested *A. fragrantissima* arial part aqueous extract on sensitive and resistant melanoma cell lines and reported a significant cytotoxicity [45]. Alenad et al. tested *A. fragrantissima* leaves methanolic extract against K562 (myeloid leukemia), Jurkat (T-cell leukemia), and HepG2 (hepatocarcinoma) cells and reported drastic morphological changes and viability reduction in K562 cells [46]. Binbreak et al. treated HepG2, A549 (lung carcinoma), HCT-116 (colorectal carcinoma), and MCF-7 (hormone-dependent breast cancer) cells with methanolic extract of *A. fragrantissima* leaves and reported cytotoxic bioactivity of the extract against A549 cells with induction of apoptosis, including activation of caspase-3, increased p53 and Bax expression, and down-regulation of Bcl-2 expression [47]. Several studies reported the isolation of *A. fragrantissima* phytochemicals and performed further experiments for the evaluation of their anticancer properties using several cell lines. For example, Awad et al. isolated several phenolic compounds, including piceol, eupatilin 7-methyl ether, chrysosplenol D, cirsiliol, and cirsimaritin, from *A. fragrantissima* leaves methanolic extract tested on MCF-7, HepG2, A549, HeLa (cervix adenocarcinoma), and PC3 (prostatic adenocarcinoma) cell lines and presented IC_50_ values ranging from 3.2 to 28.3 µg/mL [48]. To the best of our knowledge, the present study is the first to determine the in vitro cytotoxicity of different *A. fragrantissima* plant parts (i.e., flowers, leaves, and roots) crude extracts against TNBC using MDA-MB-231 cell line and to investigate the pro-apoptotic effect of *A. fragrantissima* flower dichloromethane extract.

Our experimental observations suggested that *A. fragrantissima* possesses promising anticancer activity against TNBC. After chemical analysis of the *A. fragrantissima* flower dichloromethane extract, we attempted to correlate the observed experimental findings with the identified metabolites. Several computer-aided drug discovery (CADD) approaches were utilized to provide accurate and fast predictions of several biological and pharmaceutical properties required for the further development of novel drug entities [49,50]. Therefore, PASS online webserver was utilized to validate the anticancer activity of *A. fragrantissima* identified metabolites. Interestingly, all fourteen identified metabolites demonstrated moderate to high anticancer activity confirming the observed experimental results. Moreover, the most probable molecular targets for the identified metabolites were predicted and the analysis suggested that certain enzymes and several other targets could contribute to the observed activity.

An additional molecular docking study was performed to assess the possibility of the identified metabolites including flavonoids, to bind and inhibit Tubulin [30,31,32] and CA IX enzyme [33,34]. The selection of these two targets was based on the previously reported in vitro studies that confirmed the inhibition by flavonoids, particularly, Quercetin. It is worth noting that the CA IX enzyme is highly expressed in the TNBC MDA-MB-231 cell line, which is reported to contribute to the aggressiveness and dysfunction of this type of cancer [51,52,53]. Our docking results were consistent with the previously reported studies in which Metabolite 11 (**M11**, Quercetin) possesses the highest docking score among the other metabolites against the two targets, suggesting the validity of the protein selection. Moreover, it has been demonstrated by several studies that inhibition of Tubulin polymerization and CA IX enzyme serve as a new and novel mechanism for promising anticancer activity [54,55,56,57,58,59,60,61,62].

One of the main aspects of drug design and development is to assess and evaluate the pharmacokinetics or drug-likeness properties relative to Lipinski’s rule of five required for orally active drugs [63]. Twelve out of fourteen metabolites passed the rules indicating a good pharmaceutical profile. Additionally, one of the main mechanisms for drug–drug interactions has resulted from the inhibition of one or several CYP isoenzymes involved in the metabolism of drugs. Therefore, the inhibition profile for the fourteen metabolites was evaluated and our results demonstrated that not all the metabolites inhibited the CYP isoenzymes, except for a few that were predicted to inhibit CYP1A2, CYP3A4, CYP2D6, and CYP2C9. The toxicity of the identified metabolites demonstrated immunotoxicity for six metabolites, while none were predicted to exhibit hepatotoxicity and cytotoxicity.

## 4. Materials and Methods

### 4.1. Chemicals and Reagents

Dulbecco’s Modified Eagle Medium (DMEM) plus GlutaMax-1 (4.5 g/L D-Glucose, 25 mM HEPES, Pyruvate), fetal bovine serum (FBS), TrypLE™ Express, and Dulbecco’s phosphate-buffered saline (PBS) were provided by Gibco^®^ (Waltham, MA, USA). Invitrogen NP-40 cell lysis buffer was purchased from Thermo Fisher Scientific (Carlsbad, CA, USA). Staurosporine (STS) was procured from Santa Cruz Biotechnology (Dallas, TX, USA). Methanol and ethanol were obtained from Honeywell Riedel-de Haen (Seelze, Germany) and Merck (Kenilworth, NJ, USA). Formic acid, dichloromethane, and chloroform were obtained from Sigma-Aldrich (St. Louis, MO, USA). Dimethyl sulfoxide (DMSO) was procured from Calbiochem (San Diego, CA, USA). The solvents are chromatography-grade or equivalent. Saudi Industrial Gas (Dammam, Saudi Arabia) provided purified carbon dioxide (CO_2_) gas. Ultra-pure water was produced using a Millipore (Billerica, MA, USA) system with a resistivity reading of 18.2 MΩ·cm at 25 °C.

### 4.2. Collection and Authentication of A. fragrantissima

The traditional herbal medicine of *A. fragrantissima* flowers, leaves, and roots was wildcrafted at Albatin (in Arabic, الباطن) dam area near Jalajil (in Arabic, جلاجل) city, Sudair (in Arabic, سدير) region, Kingdom of Saudi Arabia (coordinates; 25.6834° N, 45.4592° E), and authenticated by a Professor of Botany and Taxonomy. The plant parts were rinsed with filtered water and left to dry under a stream of slightly warm dry air. Once completely dried, the plant parts were fine powdered using an electric-motor grinder and kept in dark at room temperature until extraction. A voucher sample was deposited at King Saud University (Herbium, Department of Botany and Microbiology) with accession number 24575 dated on 17 June 2021 (see Supplementary Figure S1).

### 4.3. Extraction of A. fragrantissima Plant Parts and Endotoxin Removal

Approximately 500 mg of each dried *A. fragrantissima* flowers, leaves, and roots were extracted separately using 10 mL of high-purity methanol, ethanol, dichloromethane, and chloroform under high-power sonication using a Sonics (Newton, CT, USA) Vibra-Cell™ Ultrasonic Liquid Processor (Model GEX-130 probe-sonicator) for 30 min. The sonicated extract was filtered using a Sartorius stedim biotech (Göttingen, Germany) quantitative ashless paper filter under gravity-flow and dried in an incubator set at 40 °C. The remaining dried pellet residue was weighted and reconstituted with 100 to 300 µL of DMSO by vortex until completely dissolved. The reconstituted extract was stored at cool temperature in the dark until use. The averaged extraction yield percentage for the flowers-derived methanol, ethanol, dichloromethane, and chloroform solvent extracts were 30.3 (±4.2), 28.4 (±1.8), 13.4 (±0.6), and 22.5 (±1.3), respectively, the leaves-derived extracts were 34.3 (±5.1), 29.9 (±2.3), 18.9 (±4.0), and 30.2 (±2.1), respectively, and the roots-derived extracts were 9.5 (±1.1), 5.5 (±1.1), 2.4 (±0.2), and 7.7 (±0.9), respectively. The levels of endotoxins in the *A. fragrantissima* sonicated aqueous extracts were determined using the Thermo Scientific™ Pierce™ Chromogenic Endotoxin Quant kit as per the manufacturer’s instruction and as described in our previous publication [64].

### 4.4. Chromatographic Fingerprinting of A. fragrantissima Extracts

The fingerprinting of *A. fragrantissima* extracts was performed using our previously published method with modifications [65]. The major components of *A. fragrantissima* were separated using an Agilent (Agilent Technologies Inc., Santa Clara, CA) 1260 infinity HPLC-UVD and a C-18 Kinetex^®^ (250 × 4.6 mm, 5 µm particle size) column (Phenomenex Inc., Torrance, CA, USA). The multi-wave UVD was set at wavelengths ranging from 225 to 375 nm at 25 nm segments without reference. The gradient programming of methanol and ultra-pure water was as follows: 5 to 100% methanol, (0 to 35 min), 100% methanol, (35 to 40 min), and 100 to 5% methanol, (40 to 45 min) at flow rate of 1 mL/min. The injection volume was 5 µL at ambient temperature and the total run time was 45 min.

### 4.5. TNBC MDA-MB-231 Cell Culture and Treatment

The TNBC MDA-MB-231 (catalog number, HTB-26) cell line was provided by American Type Culture Collection Inc. (Manassas, VA, USA). The cells were cultured in complete DMEM Glutamax-I medium containing 10% heat-inactivated FBS, 1% penicillin G (100 IU/mL), and streptomycin (100 µg/mL) solution. The incubation was set at 37 °C in 5% CO_2_ and 95% relative humidity. The culture medium was changed every two to three days and sub-cultured when the cell population density reached 70 to 80% confluency. The cells were exposed to various concentrations of *A. fragrantissima* extracts and the appropriate controls; including 1% DMSO (negative control) and 1 µM STS (potent apoptosis inducer) were used.

### 4.6. Determination of MDA-MB-231 Cell Viability Using the CellTiter-Glo™ Assay

The cell viability was determined using Promega (Madison, WI) CellTiter-Glo™ assay according to the manufacturer’s instructions as described in [66]. Briefly, the MDA-MB-231 cells (400/cm^2^) were seeded in opaque white bottom 96-well plates (Costar, Thermo Fisher Scientific, Waltham, MA, USA). The next day, the untreated cells and the cells treated with various concentrations (0.05–2000 µg dry extract weight/mL) of each solvent extract were incubated for 48 h. Then, the plate was left at room temperature for 30 min and 100 µL of CellTiter-Glo™ reagent were added to each well and mixed on a shaker for 2 min. Luminescence was read using the Perkin Elmer^®^ (Waltham, MA, USA) EnVision^®^ multilabel plate reader.

### 4.7. Evaluation of the Hallmarks of Apoptosis

The shrinkage of cells was observed using a ZEISS inverted phase-contrast microscope (Peine, Germany). The activity of pro-apoptotic executioner caspase-3/7 and of the mitochondrial permeability transition pore opening in the untreated and treated cells were assessed using Image-iT™ LIVE Red caspase-3/7 detection kit and Image-IT™ LIVE Mitochondrial Transition Pore Assay Kit (Invitrogen, Thermo Fisher Scientific, Waltham, MA, USA), respectively, according to the manufacturer’s instructions and as previously described in [67]. The cells with activated caspase-3/7 (red fluorescence) and apoptotic cells (whole green fluorescence) with mitochondrial outer membrane permeabilization were observed under the confocal microscopy. The abnormal phosphatidylserine exposure was determined using the Becton Dickinson (BD) Annexin V apoptosis detection kit (Becton Dickinson, San Jose, CA, USA) as described in [68]. The apoptotic status of 10,000 cells based on the dual staining of Annexin V conjugated to FITC and PI conjugated to PE was analyzed using BD FACSDiva™ 6.0 software on the BD FACSCanto™ II flow cytometer.

### 4.8. Metabolite Identification Using ESI-LC-QTOF

The method of separation and identification of *A. fragrantissima* flower metabolites in dichloromethane extract was performed using an Agilent (Santa Clara, CA, USA) 1260 Infinity HPLC system coupled to Agilent 6530 quadrupole time-of-flight (Q-TOF) as described earlier in [69]. Briefly, the separation was performed using an Agilent SB-C18 column (4.6 mm × 150 mm, 1.8 μm) with the following elution gradient; 0 to 2 min, 5% B; 2 to 17 min, 5 to 100% B; 17 to 21 min, 95% B; 21 to 25 min, 5% B, using mobile-phase A (0.1% formic acid in water) and mobile-phase B (0.1% formic acid in methanol). The flow rate was set at 250 μL/min and injection volume was 10 μL. The scanning range was set at 50–800 (*m*/*z*), gas temperature at 300 °C gas flow of 8 L/min, nebulizer pressure of 35 psi, sheath gas temperature at 350 °C, and sheath gas flow rate of 11 L/min. The data were generated by the Agilent MassHunter (version B. 06.00) qualitative analysis software.

### 4.9. Anticancer Activity Predictions

The activity spectra for substances (PASS) online service provides online predictions of a variety of biological activities including anticancer predictions (http://www.way2drug.com/passonline/info.php, accessed on 25 April 2020). The Simplified Molecular Input Line Entry System (SMILES) of the chemical structures of the identified metabolites was utilized to generate the predictions using a database containing 250,000 biological activities. The generated results are represented as P_a_ (probability of active molecule) and P_i_ (probability of inactive molecule), ranging from 0.00 to 1.00. The higher P_a_ value for a certain biological activity indicates the molecule is predicted to be highly active experimentally P_a_ > 0.7), while P_a_ ranging from 0.5 to 0.7 suggests a moderate probability for experimental activity, and P_a_ lower than 0.5 indicates negligible experimental activity.

### 4.10. Molecular Target Predictions

The biological targets for the identified metabolites were predicted using the Cheminformatics tool Molinspiration webserver (https://www.molinspiration.com/, accessed on 25 April 2020). Each metabolite was evaluated as GPCR Ligand, Ion Channel modulator, Kinase Inhibitor, Nuclear Receptor Ligand, Protease Inhibitor, and Enzyme Inhibitor. The bioactivity scores were classified into three main classifications, including active (score above 0.00), moderately active (−0.50 to 0.00), and inactive (score below −0.50).

### 4.11. Molecular Docking

The molecular docking study was performed using Maestro Schrödinger software (Release 2022-1). The crystal structures of the Tubulin-Colchicine complex (Protein Data Bank identifier, PDB ID 4O2B) and human CA IX in complex with 5-[1-(4-Methylphenyl)-1H-1,2,3-triazol-4yl]thiophene-2-sulfonamide (PDB ID Y0R) were downloaded from the protein data bank (https://www.rcsb.org/, accessed on 25 April 2020). The proteins were prepared using the protein preparation wizard followed by receptor grid generation using the Glide tool. The 2D structures of the identified metabolites were converted into 3D and minimized using the LigPrep tool. All the prepared metabolites were docked using the Glide tool and the 2-D interaction map with docking scores was used to rank the docked metabolites into the two targets.

### 4.12. ADME Properties Predictions

The online accessible webserver SwissADME (http://www.swissadme.ch/, accessed on 25 April 2020) and commercial QikProp (Schrödinger software, Release 2022–1) tools were utilized to predict the ADME properties for the identified metabolites. Several important pharmaceutical properties were selected for evaluation including Molecular Weight (g/mol), Log Po/w (lipophilicity), Log S (solubility), BBB Permeability, GI Absorption, and violations of ROF.

### 4.13. CYP Enzyme Inhibition Predictions

To evaluate if the identified metabolites are substrates for the CYP enzymes, the SwissADME tool was used to evaluate the inhibition profile against five main isoenzymes (CYP1A2, CYP2C19, CYP2C9, CYP2D6, and CYP3A4). The predicted results could enrich the literature regarding the possible drug–drug interactions, toxicity, or unwanted side effects of the metabolites that result from the inhibition of CYP enzymes.

### 4.14. Organ Toxicity and Safety Predictions

The prediction of various toxicity endpoints was performed using a freely accessible webserver ProTox-II (https://tox-new.charite.de/protox_II/, accessed on 25 April 2020). For each metabolite, several toxicity endpoints were evaluated including hepatotoxicity, cytotoxicity, carcinogenicity, mutagenicity, and immunotoxicity. The ProTox-II predicted results were based on reported in vitro and in vivo assays.

### 4.15. Data and Statistical Analyses

For the cell viability experiments, the extract concentration versus inhibition curves were obtained from plotting the log_10_ treatment concentrations versus the percentage inhibition of MDA-MB-231 cell viability. The IC_50_ values were extrapolated from 50% maximum viability inhibition. The inhibition and effective doses were measured using the log-inhibition variable-slop (three or four-parameter) model in GraphPad Prism (San Diego, CA, USA) software version 5.04. For the FLICA caspase-3/7 and mitochondrial membrane transition pore opening activation assay, several images were counted for each individual treatment and averaged. Student’s *t*-test and one-way ANOVA statistical analysis were used to determine if the treatment is statistically significant to no treatment when *p* value is less than 0.05. The measured variables in this study are expressed as mean ± SD.

## 5. Conclusions

This study demonstrated the anticancer properties of *A. fragrantissima* flower dichloromethane extract through apoptosis induction using a series of in vitro apoptosis-related experiments on TNBC MDA-MB-231 cells. The findings revealed caspase-dependent apoptosis as the mechanism of cell death with the involvement of the intrinsic mitochondrial apoptosis pathway. Using in silico approaches, fourteen metabolites were extensively evaluated for their anticancer activity, which could be correlated to several mechanisms involving CA IX and Tubulin polymerization inhibition. Molecular docking investigations revealed that Quercetin was the most active hit compared to other evaluated metabolites that were consistent with previously reported studies with moderate CYP enzyme inhibition and toxicity. These findings suggest that *A. fragrantissima* flower dichloromethane extract is a promising source containing anticancer agents that could be further structurally modified and optimized to discover novel drug entities. Further in vitro and in vivo studies are required to isolate and characterize the phytochemicals and determine the potential bioactive compound(s) against TNBC.

## Figures and Tables

**Table 1:** The half-maximal inhibitory concentrations (IC_50_) of *A. fragrantissima* plant part extracts on TNBC MDA-MB-231 cell viability using the ATP-based Promega CellTiter-Glo™ assay. Results are expressed as mean ± standard deviation (SD) from at least three separate experiments.

Plant Part	Extraction Solvent	Inhibition Values (IC_50_) (µg/mL)
		Mean ± SD
Flowers		
	Methanol	122.97 ± 24.50
	Ethanol	66.77 ± 31.11
	Dichloromethane	32.43 ± 3.41
	Chloroform	33.13 ± 7.20
Leaves		
	Methanol	82.66 ± 3.88
	Ethanol	99.71 ± 9.24
	Dichloromethane	36.67 ± 3.94
	Chloroform	36.48 ± 3.02
Roots		
	Methanol	103.85 ± 27.02
	Ethanol	161.77 ± 45.59
	Dichloromethane	120.50 ± 17.15
	Chloroform	52.24 ± 11.63

**Table 2:** The bioactivity scores of *A. fragrantissima* flower dichloromethane extract-derived metabolites using PASS online webserver.

Anti-Carcinogenic Activity	Probability of Being Active (P_a_)	Probability of Being Inactive (P_i_)
**M1**	0.926	0.002
**M2**	0.831	0.008
**M3**	0.941	0.004
**M4**	0.819	0.010
**M5**	0.641	0.011
**M6**	0.867	0.005
**M7**	0.657	0.034
**M8**	0.634	0.003
**M9**	0.783	0.014
**M10**	0.461	0.083
**M11**	0.797	0.012
**M12**	0.575	0.014
**M13**	0.445	0.068
**M14**	0.905	0.005

**Table 3:** Predicted biological targets of *A. fragrantissima* flower dichloromethane extract-derived metabolites using Molinspiration webserver.

Compound Name	Target Predictions Using Molinspiration	
**M1**	GPCR ligand Ion channel modulator Kinase inhibitor Nuclear receptor ligand Protease inhibitor Enzyme inhibitor	0.10 −0.01 0.14 0.31 0.02 0.43
**M2**	GPCR ligand Ion channel modulator Kinase inhibitor Nuclear receptor ligand Protease inhibitor Enzyme inhibitor	−0.15 −0.28 0.14 0.01 −0.35 0.13
**M3**	GPCR ligand Ion channel modulator Kinase inhibitor Nuclear receptor ligand Protease inhibitor Enzyme inhibitor	0.31 0.17 −0.08 0.81 0.21 0.81
**M4**	GPCR ligand Ion channel modulator Kinase inhibitor Nuclear receptor ligand Protease inhibitor Enzyme inhibitor	−0.09 −0.23 0.20 0.13 −0.29 0.14
**M5**	GPCR ligand Ion channel modulator Kinase inhibitor Nuclear receptor ligand Protease inhibitor Enzyme inhibitor	−0.07 −0.09 0.18 0.34 −0.25 0.26
**M6**	GPCR ligand Ion channel modulator Kinase inhibitor Nuclear receptor ligand Protease inhibitor Enzyme inhibitor	−0.03 0.02 −0.48 0.34 0.00 0.33
**M7**	GPCR ligand Ion channel modulator Kinase inhibitor Nuclear receptor ligand Protease inhibitor Enzyme inhibitor	−0.06 0.26 −0.78 0.37 −0.38 0.43
**M8**	GPCR ligand Ion channel modulator Kinase inhibitor Nuclear receptor ligand Protease inhibitor Enzyme inhibitor	0.17 0.21 −0.38 0.65 0.18 0.58
**M9**	GPCR ligand Ion channel modulator Kinase inhibitor Nuclear receptor ligand Protease inhibitor Enzyme inhibitor	−0.02 −0.07 0.26 0.39 −0.22 0.28
**M10**	GPCR ligand Ion channel modulator Kinase inhibitor Nuclear receptor ligand Protease inhibitor Enzyme inhibitor	−0.06 −0.07 −0.62 0.09 −0.04 0.24
**M11**	GPCR ligand Ion channel modulator Kinase inhibitor Nuclear receptor ligand Protease inhibitor Enzyme inhibitor	−0.06 −0.19 0.28 0.36 −0.25 0.28
**M12**	GPCR ligand Ion channel modulator Kinase inhibitor Nuclear receptor ligand Protease inhibitor Enzyme inhibitor	0.78 0.17 0.27 −1.52 −0.28 0.86
**M13**	GPCR ligand Ion channel modulator Kinase inhibitor Nuclear receptor ligand Protease inhibitor Enzyme inhibitor	0.27 0.10 −0.12 0.22 0.14 0.20
**M14**	GPCR ligand Ion channel modulator Kinase inhibitor Nuclear receptor ligand Protease inhibitor Enzyme inhibitor	0.12 −0.04 −0.52 0.69 0.08 0.64

**Table 4:** Docking scores of identified metabolites with Tubulin crystal structure.

Compound Name	Docking Score (kcal/mol)	Interactions with Amino Acid Residues
**M1**	−9.59	Lys254, and Val181
**M2**	−9.16	Asp251, and Glu183
**M3**	−6.90	Thr353, Cys241, and Val238
**M4**	−8.60	Asp251, and Cys241
**M5**	−8.10	Asp251, Cys241, and Asn350
**M6**	−7.07	Lys254
**M7**	−4.75	Cys241, and Val238
**M8**	-	-
**M9**	−8.43	Val181, Cys241
**M10**	−6.38	Asp251, Cys241
**M11**	−9.89	Cys241, Val181, and Asn350
**M12**	−7.35	Val181, Cys241
**M13**	−4.23	Val238
**M14**	−7.40	Thr179, Val181
**Colchicine**	−11.35	Val181, Cys241, Thr353, Val238

**Table 5:** Docking scores of identified metabolites with CA IX crystal structure.

Compound Name	Docking Score (kcal/mol)	Interactions with Amino Acid Residues
**M1**	−5.01	Thr200, Thr201, Gln92, Leu91, and zinc coordination
**M2**	−4.08	Asn66, and zinc coordination
**M3**	−4.14	Gln92
**M4**	−5.25	Asn66, Thr200, Zinc coordination
**M5**	−5.21	His68, Thr200, and zinc coordination
**M6**	−4.07	Gln92, and zinc coordination
**M7**	−3.32	Gln92
**M8**	-	-
**M9**	−5.20	Zinc coordination
**M10**	-	-
**M11**	−5.46	His68, Thr200, His94, Zinc coordination
**M12**	−4.52	His68, Thr201, and zinc coordination
**M13**	−1.30	Gln92, His94, Thr200, and zinc coordination
**M14**	−4.40	Gln71, Thr201, Gln92, and zinc coordination
**Y0R**	−7.13	Gln92, Thr200, and zinc coordination

**Table 6:** The predicted ADME properties of *A. fragrantissima* flower dichloromethane extract-derived metabolites using SwissADME webserver.

Compound Name	Molecular Weight (g/mol)		Log Po/w		Log S		BBB Permeant		GI Absorption		Rule of Five (ROF)
	SWISS ADME	QikProp	SWISS ADME	Qik Prop	SWISS ADME	Qik Prop	SWISS ADME	Qik Prop	SWISS ADME	Qik Prop (%)	SWISS ADME
**M1**	432.38	432.38	0.05	−0.36	−2.69 Soluble	−3.22	No	No	Low	29.50	Yes; 1 violation: NHorOH > 5
**M2**	360.31	360.32	2.60	2.21	−4.74 Moderately soluble	−3.92	No	No	High	82.42	Yes; 0 violation
**M3**	336.34	336.34	0.82	1.66	−1.52 Soluble	−3.466	No	No	High	82.33	Yes; 0 violation
**M4**	344.32	344.32	2.90	2.79	−5.33 Moderately soluble	−4.142	No	No	High	92.79	Yes; 0 violation
**M5**	270.24	270.24	2.58	1.59	−4.40 Moderately soluble	−3.297	No	No	High	73.11	Yes; 0 violation
**M6**	306.35	306.35	1.90	2.34	−2.27 Soluble	−3.888	Yes	Yes	High	92.97	Yes; 0 violation
**M7**	222.37	222.37	4.23	4.60	−3.00 Soluble	−4.95	Yes	Yes	High	100	Yes; 0 violation
**M8**	436.62	436.63	3.93	3.80	−3.39 Soluble	−5.759	No	No	High	95.21	Yes; 0 violation
**M9**	286.24	286.24	2.28	0.91	−3.82 Soluble	−3.026	No	No	High	61.10	Yes; 0 violation
**M10**	497.71	497.71	6.54	6.40	−6.30 Poorly soluble	−8.969	No	No	High	100	Yes; 1 violation: MLOGP > 4.15
**M11**	302.24	302.24	1.99	0.353	−3.24 Soluble	−2.882	No	No	High	51.56	Yes; 0 violation
**M12**	282.25	282.25	−2.58	−1.68	0.59 Soluble	−1.693	No	No	Low	49.49	Yes; 0 violation
**M13**	287.40	287.40	3.74	4.28	−4.81 Moderately soluble	−5.433	Yes	No	High	100	Yes; 0 violation
**M14**	278.30	278.30	1.10	0.28	−2.52 Soluble	−2.155	No	No	High	68.622	Yes; 0 violation

**Table 7:** The CYP-P450 enzyme inhibition profile of *A. fragrantissima* flower dichloromethane extract-derived metabolites using SwissADME webserver.

Compound	CYP1A2	CYP2C19	CYP2C9	CYP2D6	CYP3A4
**M1**	No	No	No	No	No
**M2**	Yes	No	Yes	Yes	Yes
**M3**	No	No	No	No	No
**M4**	Yes	No	Yes	Yes	Yes
**M5**	Yes	No	No	Yes	Yes
**M6**	No	No	No	No	No
**M7**	No	No	Yes	No	No
**M8**	No	No	No	No	No
**M9**	Yes	No	No	Yes	Yes
**M10**	No	No	Yes	No	No
**M11**	Yes	No	No	Yes	Yes
**M12**	No	No	No	No	No
**M13**	Yes	Yes	Yes	No	Yes
**M14**	No	No	No	No	No

**Table 8:** Organ and endpoint toxicity predictions of *A. fragrantissima* flower dichloromethane extract-derived metabolites using the ProTox-II webserver.

Compound Name	Classification				
	Organ Toxicity (% Probability)	Toxicity Endpoint (% Probability)			
	Hepatotoxicity	Carcinogenicity	Immunotoxicity	Mutagenicity	Cytotoxicity
**M1**	0.82 (inactive)	0.86 (inactive)	0.93 (inactive)	0.59 (active)	0.69 (inactive)
**M2**	0.70 (inactive)	0.69 (inactive)	0.97 (Active)	0.82 (inactive)	0.75 (inactive)
**M3**	0.67 (inactive)	0.61 (inactive)	0.99 (Active)	0.55 (Active)	0.58 (inactive)
**M4**	0.70 (inactive)	0.69 (inactive)	0.84 (Active)	0.82 (inactive)	0.75 (inactive)
**M5**	0.68 (inactive)	0.62 (inactive)	0.99 (inactive)	0.57 (inactive)	0.87 (inactive)
**M6**	0.72 (inactive)	0.54 (Active)	0.94 (Active)	0.68 (inactive)	0.77 (inactive)
**M7**	0.78 (inactive)	0.70 (inactive)	0.97 (inactive)	0.83 (inactive)	0.75 (inactive)
**M8**	0.60 (inactive)	0.75 (inactive)	0.57 (Active)	0.73 (inactive)	0.75 (inactive)
**M9**	0.69 (inactive)	0.68 (Active)	0.97 (inactive)	0.51 (Active)	0.99 (inactive)
**M10**	0.81 (inactive)	0.52 (inactive)	0.98 (Active)	0.79 (inactive)	0.75 (inactive)
**M11**	0.69 (inactive)	0.68 (Active)	0.87 (inactive)	0.51 (Active)	0.99 (inactive)
**M12**	0.68 (inactive)	0.73 (inactive)	0.97 (inactive)	0.83 (inactive)	0.62 (inactive)
**M13**	0.83 (inactive)	0.62 (inactive)	0.84 (Active)	0.76 (inactive)	0.83 (inactive)
**M14**	0.71 (inactive)	0.63 (inactive)	0.71 (inactive)	0.84 (inactive)	0.64 (inactive)

## Data Availability

The data presented in this study are available on request from the corresponding author.

## Supplementary Materials

The following supporting information can be downloaded at https://www.mdpi.com/article/10.3390/ph15091060/s1, Figure S1: Authentication of *A. fragrantissima* flower. Deposited voucher sample.

## Abbreviations

ADME	absorption, distribution, metabolism, and excretion
BBB	blood–brain barrier
BD	Becton Dickinson
CA IX	carbonic anhydrase IX
CADD	computer-aided drug discovery
CoCl_2_	cobalt chloride
CO_2_	carbon dioxide
CYP	cytochrome P450
DNA	deoxyribonucleic acid
DMEM	Dulbecco’s Modified Eagle Medium
DMSO	dimethyl sulfoxide
EC_50_	half-maximal effective concentration
EU	endotoxin unit
FACS	fluorescence-activated cell sorting
FBS	fetal bovine serum
FITC	fluorescein isothiocyanate
FLICA	fluorochrome-labeled inhibitors of caspases
FMK	fluoromethyl ketone
GI	gastrointestinal
GPCR	G protein-coupled receptor
HPLC-UVD	high-performance liquid chromatography and ultra-violet detector
IC_50_	half-maximal inhibitory concentration
log_10_	common logarithm (base 10)
PASS	prediction of activity spectra for substances
PBS	phosphate-buffered saline
PDB ID	Protein data bank identifier
PE	phycoerythrin
PI	propidium iodide
QTOF	quadrupole time-of-flight
ROF	rule of five
SD	standard deviation
THM	traditional herbal medicine
TNBC	triple-negative breast cancer
