# Supplementary figures and images for: Achillea fragrantissima (Forssk.) Sch.Bip Flower Dichloromethane Extract Exerts Anti-Proliferative and Pro-Apoptotic Properties in Human Triple-Negative Breast Cancer (MDA-MB-231) Cells: In Vitro and In Silico Studies
Source: Pharmaceuticals (Basel). 2022 Aug 26;15(9):1060. doi: 10.3390/ph15091060 (PMC9506496; doi:10.3390/ph15091060)

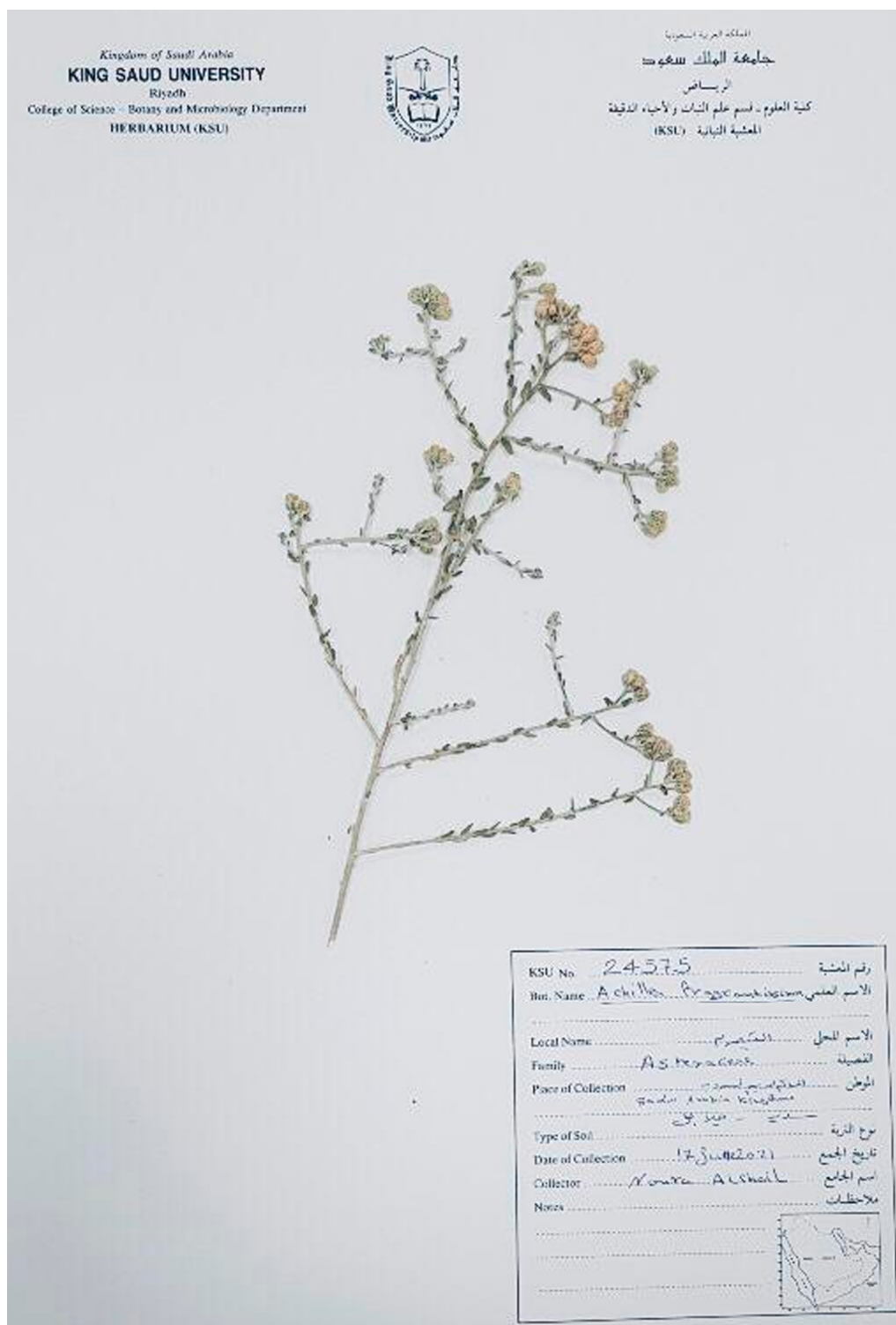

**Figure S1:** Authentication of *A. fragrantissima* flower. Deposited voucher sample.

Supplement: Supplementary file 1 [file pharmaceuticals-15-01060-s001.zip › pharmaceuticals-1752329-supplementary.pdf]
